# A mobile application to protect groundwater during unconventional oil and gas extraction

**DOI:** 10.1098/rsos.220221

**Published:** 2022-09-14

**Authors:** Charissa Worthmann, Surina Esterhuyse

**Affiliations:** Centre for Environmental Management, University of the Free State, P.O. Box 339, Bloemfontein, Free State 9301, South Africa

**Keywords:** unconventional oil and gas (UOG), hydraulic fracturing (fracking), regulatory enforcement, civic informatics, mobile application, groundwater protection

## Abstract

Unconventional oil and gas (UOG) is an important energy source for many countries, but requires large quantities of water for its development, and may pollute water resources. Regulations are one of the main tools to achieve government policy on natural resource protection. South Africa, which is energy-constrained, but also water-scarce, is currently considering UOG extraction as an additional energy resource. UOG development could commence as soon as regulations to protect natural resources such as water have been published. Such regulations are, however, often not effectively enforced, which negatively affects the protection of water resources during UOG extraction. This study addresses these enforcement challenges in South Africa. It focuses on the science–society–policy interface by proposing a civic informatics platform to assist with on-the-ground enforcement of regulations via a mobile application. This mobile application aims to address both groundwater monitoring and management as well as UOG extraction operations in a single platform, to enable regulators to protect groundwater resources more effectively during UOG extraction, while simultaneously enhancing transparency in the UOG industry.

## 1. Introduction

Globally, countries are experiencing more water stress due to climate change, industrial growth and population growth [1]. South Africa is one such country that is experiencing severe water stress and is approaching physical water scarcity [1–4]. An estimated 45.5% of the country's surface area is more than 50% dependent on groundwater supply for domestic needs [5]. Groundwater, therefore, plays a key role in the country and is required for human survival, in terms of ensuring food security, economic development and maintaining the environment [4,6,7]. Because groundwater has become a critically strained resource and due to the current extent of groundwater pollution in South Africa, the development of groundwater protection measures in South Africa requires urgent attention [7].

South Africa is, however, also energy-constrained and is currently considering unconventional oil and gas (UOG) development as an additional energy resource to improve the country's energy security [8]. South Africa's Integrated Resource Plan outlines gas production as an important contributor to diversifying its energy mix and serves as a bridge to renewables [9]. However, for South Africa to reach net-zero carbon emissions by 2050, the gas must be substituted with greener alternatives and phased out by 2050 [10]. South Africa, therefore, commissioned the Strategic Environmental Assessment of Shale Gas in the Karoo in 2016 [11] and the Petroleum Agency of South Africa commissioned the development of a groundwater monitoring baseline in the Karoo [12]. UOG extraction can, however, have serious environmental impacts, of which the most important is the impact on water resources, specifically groundwater [13]. Water and energy are interlinked resources that are crucial to socio-economic development, and their protection and sustainable use during economic development are paramount for protecting these resources for current and future generations [4].

UOG development in South Africa faces many challenges. Many South Africans doubt the potential benefits of UOG development for South Africa's energy supply or job opportunities and locals are pessimistic about the government's readiness for UOG development [14,15]. The government faces regulatory challenges because of poor interdepartmental collaboration, miscommunications, poor drafting and enforcement of regulations, and a high level of mismanagement across South Africa's intricate inter-governmental system [16]. Additionally, regulations that aimed to minimize UOG extraction impacts, were set aside by the Supreme Court of Appeal in 2015 because they did not address all the relevant environmental concerns [17]. Despite the concerns related to UOG extraction, the government is pursuing shale gas extraction [7,18]. Even though some coal-bed methane deposits (CBM) also occur in the Karoo basin, shale gas is currently the main focus for UOG resource development in South Africa [4].

Since UOG extraction is extremely water-intensive and can pollute water resources [1,19], the drafting of relevant regulations and effectively enforcing them is crucial to protect water resources. South Africa has no prior experience of onshore UOG extraction, and successful resource governance will therefore also depend on the inclusion of broader society for the generation of knowledge and applicable decision-making [20]. Stakeholders who will be impacted by and who are involved in UOG extraction are important role players in streamlining the process of regulatory compliance and successful management [21]. To address the concerns of all stakeholders during regulation, and to ensure transparency and bridge the information gap between citizens, UOG extraction companies and regulatory authorities, we propose the development of a civic informatics (CI) platform that can be implemented via a mobile application. A CI platform is a technological system that uses public and institutional data to address environmental management problems such as the regulation of UOG extraction and is an ideal tool to serve as an interface between science, society and policy. Since South Africa is highly dependent on groundwater, our study focused on the development of a CI platform to properly enforce fracking regulations to protect groundwater resources [22].

## 2. Methods

We designed a framework for a mobile application that should enable better enforcement of fracking regulations to protect specifically groundwater resources. Such a framework would be a useful tool for enforcing regulations in South Africa and other countries with a high dependency on groundwater resources.

To design the framework, we systematically reviewed scholarly literature to:

— Identify the possible negative effects of UOG extraction on groundwater resources.
— Assess international regulatory shortcomings on groundwater resources protection during UOG extraction.
— Review the international use of CI and its related use within mobile and/or desktop applications to assist with the enforcement of fracking regulations. Existing UOG extraction mobile applications and the information they provide were also reviewed.
— The above information was used to develop the structure of the proposed mobile application.
— We used this information to develop the structure of the proposed mobile application to protect groundwater resources during UOG extraction in South Africa. We did this by contextualizing UOG extraction in South Africa and by reviewing potential CI platforms and stakeholders that can address UOG extraction in South Africa and mapping relationships between parties.

For the literature review, we used Google Scholar, the Web of Science, HeinOnline and Legal Source databases to find key articles on the impacts of fracking, international regulatory and monitoring approaches, the use of mobile applications and interactive websites to manage UOG extraction information and information on the history of fracking in South Africa. Backward and forward snowballing was used to identify additional relevant literature sources from the articles that were identified from the databases [23]. The literature was delimited using the following criteria:

— The literature only focused on *onshore* oil and gas extraction because this study focuses on managing UOG extraction groundwater impacts experienced on land.
— We only reviewed literature that addressed groundwater-related impacts and not the other environmental, social or economic impacts posed by UOG extraction.
— For the assessment of possible groundwater impacts of UOG extraction, we reviewed studies from countries where UOG extraction is already practised, which mostly included developed countries and very limited examples from developing countries. Although developed countries were used in this study, the experiences from these countries were considered in the context of a developing country.

Using the above information, we propose a framework for the development of a mobile application that will enhance the governance of UOG extraction and foster cooperation between the relevant stakeholder groups in South Africa, to ensure the flow of key information and better enforcement of UOG extraction regulations to protect groundwater resources.

## 3. Results

### 3.1. Possible negative effects of unconventional oil and gas extraction on groundwater resources

Commonly targeted UOG resources include shale gas and CBM. During the process of UOG extraction, the use of groundwater, the actual fracking operations, the generation of wastewater and solid waste management and the decommissioning of production wells can all impact groundwater resources negatively. The effects of each of these aspects of UOG extraction on groundwater resources are individually discussed below.

#### 3.1.1. Groundwater use

Fracking, which requires large volumes of water, is used in UOG production to release the gas and is used specifically in shale gas extraction. Fracking can, however, also be used in CBM extraction if depressurization methods do not release the trapped gas effectively [5]. In Australia, fracking is often used together with other gas stimulation methods during CBM development, and in the future, fracking could be used in as much as 40% of CBM wells [24]. UOG production and the hydraulic fracturing process are water-intensive [25], with a single well requiring anything from 5 to 11 million gallons (19–42 million litres) of water [26]. In sweet-spot oil-producing areas, oil producers use water even more intensively [27]. This intensive water use is seen worldwide, for example in the Permian Basin in Texas and New Mexico, the wells using at least 400 thousand barrels of water per completion have risen from 0.3% in 2013 to 43% in 2016 and 60% in 2017 [27].

Most of the water is used during the drilling and fracturing process. Fracking fluid is created by mixing water with sand and chemicals [15]. After hydraulic fracturing, this fracking fluid returns to the surface as flowback water and is no longer a viable potable water resource and is disposed of in salt or brine disposal wells [26]. Flowback is rarely re-used [19] because reuse of this water can damage equipment and compromise the future success of UOG extraction [26]. New freshwater sources are therefore continuously targeted for drilling and fracturing processes. This is especially concerning in arid areas, as these freshwater sources are usually drawn from sources close to the fracking well, resulting in the large abstraction of groundwater resources for fracking. Consequently, the extraction of groundwater for use during fracking operations can cause groundwater drawdown and affect aquifer structural integrity, possibly reducing the aquifer's storage capacity [28].

In water-stressed regions, new freshwater abstraction can have adverse consequences. In the United States (US), irrigation constitutes approximately 33% of water use, of which 83% is used in the western states for agriculture. During the 2011 drought, the US Texan government prioritized water allocation for fracking over the allocation for agriculture, ultimately resulting in community uproar and litigation measures. This signals that water conflicts in arid UOG extraction areas will potentially become more common in the future [29].

#### 3.1.2. Fracking operations

Fracking operations can negatively affect the integrity of an aquifer by changing its geological structure. Natural preferential pathways (such as faults) or man-made preferential pathways (such as oil and gas wells) can lead to the contamination of freshwater aquifers when upward migration of fracking fluids from the shale reservoir occurs along these pathways [2].

#### 3.1.3. Wastewater and solid waste management

Aquifer contamination is likely to occur when there is inadequate solid waste or wastewater management [2]. The wastewater produced during shale gas extraction and CBM can differ, with flowback from fracking fluids making up a large component of shale gas extraction wastewater, while produced water emanating from the geological formations is the more common wastewater type in the case of CBM. If CBM wells are fracked, they will also produce flowback. Produced water is generated for both shale gas and CBM operations [5]. One highlighted concern is the groundwater contamination resulting from wastewater (consisting of both flowback and produced water). In terms of flowback water, Grant and Chisholm [30] estimated that between 0 and 80% of the fracking fluid can return to the surface. Produced water is also released from geological formations over the lifetime of the well, potentially comprising radioactive material and heavy metals [31]. Pollution of surface water resources and/or the infiltration of surface contaminants into groundwater resources can also occur through the occurrence of accidental spillages (such as drilling fluid or fracking fluid) or via flowback or produced water.

With fracking wells often located in close vicinity to public drinking water sources, the risk of negative impacts on groundwater associated with fracking significantly rises for users in the region. To put this into perspective, the United States Environmental Protection Agency (US EPA) estimated that between the years 2000–2013, public water systems serving over 8.6 million people were within 1.6 km (or one mile) from a fracking well [29]. In Australia, CBM is more often produced. The CBM deposits often occur near freshwater aquifers and large volumes of wastewater are generated during gas production. The CBM extraction process itself can therefore endanger freshwater aquifers while poor wastewater management also endangers water resources [24]. These two examples demonstrate the far-reaching magnitude of water pollution.

#### 3.1.4. Decommissioning of production wells

Fracking fluid, flowback or produced water can migrate into aquifers when there is poor well design or well integrity failure. A variety of estimated percentages have been reported regarding well integrity failure, and results range between 1.9 and 3.4%. The ageing of well components may potentially increase this risk by 18%, as observed in additional well inspections [32]. The failure of disposal wells over time is one of the key causes of conflict due to the complexity of the consequences of well failure. For example, produced water that is released as a result of casing failure can cause widespread groundwater pollution [26]. However, the impacts thereof may go unnoticed or only be identified at a later stage, which complicates the assignment of liability [2]. Furthermore, environmental impacts on ecosystems do not always respond predictably. Soil and water contamination also pose a considerable risk to locals and the environment, becoming amplified in arid ecosystems and where communities are highly dependent on groundwater resources [31]. It is therefore paramount to safely decommission production wells that are no longer used and to continuously monitor the decommissioned wells to protect groundwater quality in the long term [33,34].

### 3.2. International regulatory shortcomings on groundwater resources protection before and during UOG extraction

The following regulatory shortcomings of UOG extraction are important to address in regulations that must protect groundwater resources:

*The poor UOG extraction knowledge base* of many countries must be addressed by developing an evidence base. This will allow for the robust regulation of UOG extraction, which is a concern in countries that extract large volumes of UOG, including the US [35–37], the UK [38,39], Europe [39,40] and Australia [41]. This highlights the vital importance and crucial need for baseline studies on groundwater to be performed before the extraction of UOG. Such data will provide information on water quality before UOG extraction and will allow for an analysis of potential impacts of fracking before, during and after UOG extraction [29].

*Lax water resources rules and regulations* for the protection of groundwater resources during fracking should be addressed. Lax water regulation is an international challenge and has been reported as a concern in the US [42–46], China [47,48], Australia [49] and Canada [29].

*Gaps in regulations*, such as dealing with wastewater handling, should be addressed. Examples of regulatory gaps are ample, for example in the US and Canada [50–52] and India [53]. In India, for example, only the inflow and outflow of groundwater are regulated for UOG extraction, but not groundwater recharge, excessive extraction or ecological impacts on groundwater during industrial extraction. This regulatory gap, as well as failed implementation, further exacerbates the pollution of India's groundwater, which in combination with polluted surface water is already at 70% [53].

*A lack of transparency in reporting data* on water use, wastewater generation and spillages is a concern in many countries, including the US [54–61], the UK [19,39,62], Europe [63–65] and China [66,67], and has fuelled conflict situations worldwide [29]. Inadequate data disclosure regulation can mean that an individual landowner has to prove injury or wrongdoing [29]. Often, fracking companies in the US are not legally obliged to reveal the chemical components used in their fracking fluid [29,68] due to the business risk it would initiate by revealing ‘confidential business information’ [69]. Although FracFocus—the publicly accessible national hydraulic fracturing chemical disclosure registry in the US—serves as a website that provides information on the use of fracking chemicals per well [70], the omission of certain companies' data renders the database incomplete [29]. FracFocus is an important regulatory tool for compliance in terms of chemical disclosure, but incomplete data cannot convey an accurate picture of the impacts of UOG extraction on groundwater. Companies also often disclose information and data in a manner that either bombards the public with information uninterpretable by the general reader or undermines the regulations [22,68], which makes it challenging for emergency response teams to handle accidents, spills or any emergency.

*The lack of federal regulation* is also a bone of contention in the US [50,55,71,72]. Here, each state can govern and protect its water resources as they see fit, with no standardized regulatory approach. This means that transboundary aquifer impacts from UOG extraction may go unregulated.

*Large-scale rollbacks of regulations* aimed at protecting water resources have occurred, especially in the US, to promote the UOG industry [13,73–75]. Other countries where rollbacks occurred include Canada [76], Amazonia [77,78] and Australia [78]. Such rollbacks are leaving groundwater resources unprotected.

### 3.3. Civic informatics and mobile applications as an aid for enforcing fracking regulations

In the US, the complexity and severity of the environmental impacts associated with UOG extraction, and the difficulties in regulating this industry, have led to greater public participation in citizen science and CI (see box 1). The complexity of various aspects of UOG extraction confuses public understanding and the understanding of the regulator regarding the impacts of this industry. For example, surface water, shallow groundwater and deep groundwater impacts are often all interlinked and can affect all components of the water–energy–food nexus [1,4,71]. The diffuse and regional-scale placement of UOG extraction infrastructure leads to spatial knowledge gaps that make it difficult to do comprehensive environmental assessments that accurately identify cumulative impacts [2,68,82]. With information disclosure exemptions, understanding environmental impacts and the linked health outcomes become increasingly difficult for citizens, as does finding common ground with others [22,68,79,81]. Legislation that currently governs UOG extraction in the US, does not address these different knowledge gaps [67,68,80]. For example, in Pennsylvania, UOG extraction companies are only required to notify properties that are in close range and adjacent to the wells about development plans, meaning that the rest of the public will only find out once permits have been allocated [25,68].

Box 1. **Civic informatics—an explainer.**

*Citizen science*: the approach of using volunteers who are not employed in project at hand to gather scientific data that are used by scientists and regulators [52,79,80].

*Civic informatics*: the facilitation of the flow of critical knowledge and information, based on informational practices of civic society research organizations. CI is considered vitally important for the two-way flow of information [64].

*Community information network*: a network promoting a centralized coordination of information and communication relating to a specific region of interest [81].

*Community information infrastructure*: the communication structures within a community. This includes organizational structures or various types of government and social organizational structures, public places where citizens come together to communicate, local media and libraries serving as information sources for the community, and finally, neighbourhood groups and networks [81].

With the deep lack of public understanding of the UOG industry and significantly delayed academic research, concerned citizen groups and other non-governmental organizations in the US began probing for information across a large network, called a community information network [68]. The University of Pittsburgh began a public service project where they aimed to map the UOG extraction wells and provide a platform for citizens to report regulatory violations (including visually identified impacts and health impacts) in the Pennsylvanian Marcellus Shale formation in a crowd-sourced manner [68]. This ultimately led to the establishment of the FracTracker Alliance, which aimed to unblur the inaccurate, incomplete and indigestible information provided by the government and companies to the public. In 2012, FracTracker restructured as a non-profit organization with a desktop and mobile application that covers a broader range of issues related to UOG extraction, and so became the key component for facilitating the flow of information between citizens, professionals and the regulatory authorities. FracTracker has demonstrated that the practices of civil research organizations are the key to a critical flow of information between citizens and science. This was the first instance of the use of CI in the context of fracking.

CI can be very effectively incorporated into mobile applications via a community information network. A community information network that transfers information and creates relationships and networks of trust can foster civic engagement [83–85]. When using a community information network in the form of a mobile application, the community information infrastructure (see box 1) is important. For the mobile application to be of benefit to society, the nature of the information infrastructure (the relationships between information types and providers, as well as who the stakeholders are) and the intended function of the community information network must be understood [83]. Mobile applications can include functions where citizens can log data on UOG transgressions and possible groundwater pollution [86–90]. Ideally, such data must be vetted, entered into a database to ensure accessibility and transparency, and presented as digestible information for the public and government [22].

## 4. A proposed mobile application framework for groundwater protection during unconventional oil and gas extraction in South Africa

### 4.1. Contextualizing unconventional oil and gas extraction and the South African governance system

To contextualize fracking in South Africa, we discuss the history of UOG development from the year 2009, which was the first time there was a marked interest in pursuing fracking in SA. We also briefly discuss South Africa's regulatory capacity.

**Figure 1.**
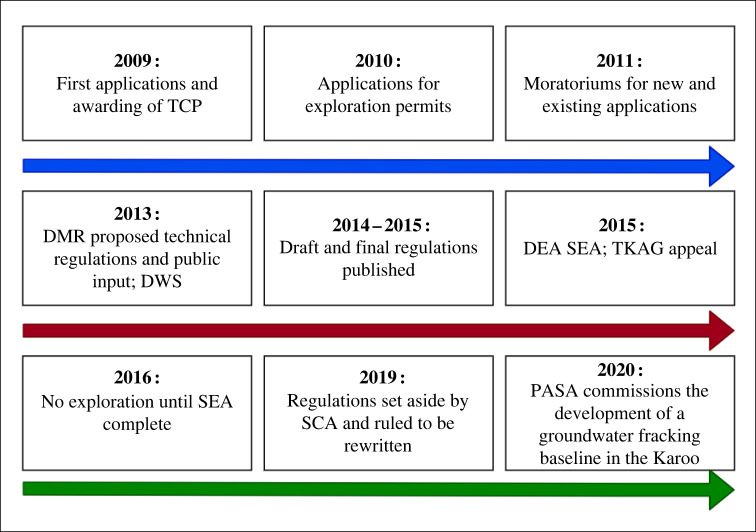
Brief chronology of UOG development in Southern Africa.

In 2009, four major companies (Bundu Gas and Oil Exploration (Pty) Ltd; Falcon Oil and Gas Ltd (Canadian) and Shell Exploration Company B.V.; and Sasol (Pty) Ltd) applied for, and were awarded, Technical Cooperation Permits under the MPRDA to pursue fracking in the Karoo with the Department of Mineral Resources [91], figure 1. The Treasure the Karoo Action Group (TKAG) subsequently requested a critical review of the exploration licence that was submitted to the Petroleum Agency of South Africa by Shell Exploration Company B.V. This critical review resulted in the instatement of two moratoriums in 2011 by the Minister of Mineral Resources [91]. These moratoriums were placed on new exploration licence applications as well as the processing of existing exploration licences, although the second moratorium was never gazetted by the government. The moratoriums were supposed to last three months, as the government launched an intergovernmental Task Team to generate a report on *Investigation of Hydraulic Fracturing in the Karoo Basin of South Africa* but ended up being in place 17 months. At the end of 2013, the DMR released their *Proposed Technical Regulations for the Exploration and Exploitation of Petroleum Resources,* for public comment. In June of 2015, the Minister of Mineral Resources published the final fracking regulations for South Africa [15,91]. In May of 2015, the Department of Environmental Affairs authorized the Council for Scientific and Industrial Research (CSIR) to perform a strategic environmental assessment (SEA) on fracking in the Karoo. The SEA assessed the opportunities and risks of shale gas extraction and proposed a decision-making framework for policymakers [11]. At the end of 2015, the TKAG and AfriForum appealed against the published fracking regulations at the Gauteng High Court (TKAG, 2011), which ruled against these parties. The case went to the Supreme Court of Appeal of South Africa (SCA) in 2019. In October 2019, the RSA SCA (2019) ruled that only the Minister of Environmental Affairs was empowered by NEMA, under the *One Environmental System (OES)*, to make regulations regarding environmental matters, and not the Minister of Mineral Resources. The SCA also stated that it was impractical to sever the invalid fracking regulations from the petroleum regulations, and the SCA, therefore, set aside the fracking regulations in their entirety. The OES is an agreement entered between the Minister of Environmental Affairs, Minister of Mineral Affairs and Minister of Water Affairs (RSA SCA, 2019) which established NEMA as the sole environmental statute and the Minister of Environmental Affairs as the responsible minister for setting the regulatory framework, norms and standards thereof [17]. To address environmental concerns related to UOG extraction in South Africa, the Petroleum Agency of South Africa commissioned the development of a groundwater monitoring baseline in the Karoo in 2020 [12].

In South Africa, the National Water Act (36 of 1998) [92] is the cornerstone of groundwater governance. South Africa's policy and strategy on groundwater quality management [93] requires its protection for current and future generations. Although the South African government is busy developing fracking regulations, a study by Esterhuyse *et al.* [22], found that enforcement, although very important, could fail for most of the proposed fracking regulations because of poor funding, weak institutions, absent legislation and regulations, political will and corruption.

To address these enforcement challenges and to ensure adherence to South Africa's groundwater policy, an independent organization with competent staff to enforce the regulations and regulatory review by independent organizations, would be required. Monitoring and compliance data should be publicly available and should be understandable by the general public. The responsible use of publicly disclosed information is also important. The CI platform proposed in this study aims to address these enforcement challenges.

### 4.2. A potential civic informatics platform to manage the regulation of unconventional oil and gas extraction

The CI platform that we propose for our mobile application must allow for the communication of key information on UOG extraction activities that can aid in the enforcement of groundwater protection regulations. It must include an external outward-facing platform that is visible to the general public and an internal platform that is visible only to the internal platform stakeholders, so that sensitive information may be protected.

Different stakeholders with appropriate competencies should play different roles on this platform. For example, several possible stakeholders can manage sections within a proposed UOG extraction CI platform. The possible platform stakeholders who can manage different sections of the platform include academia (academic institutions), parastatals, private consultancies and non-governmental organizations. This proposed structure is based on the concept of CI discussed in the previous section.

#### 4.2.1. Academia

Universities should address societal needs by adapting to changing technology, industry changes, population dynamics and changing global trends [94]. Several South African academic institutions have emerged as potentially key players in the CI arena on UOG extraction and groundwater protection. The University of the Free State developed an interactive map that maps groundwater vulnerability, borehole information and geological information [5,95]. Such a map can be used as a springboard for communicating UOG extraction on a CI platform. The North West University also recently developed a groundwater monitoring mobile application that makes use of citizen science to log the location of boreholes across the country, specifically aiming to bridge the gap in data available in the country [96]. This is an example of where a mobile application can be used for data gathering to bridge the gap between the regulatory authority (government), citizens and academia.

#### 4.2.2. Parastatals

Parastatals in South Africa, such as the Council for Geoscience or the Council for Scientific and Industrial Research (CSIR) could manage significant sections of a CI platform and play a large role in its management. The CSIR recently performed a strategic environmental assessment (SEA) for shale gas extraction in the Karoo and together with this SEA, also developed a related GIS map database on shale gas information in South Africa [11]. The map database could be used to communicate information on UOG extraction areas and provide technical information once developed. Parastatals would play a key role in ensuring the flow of critical information between the CI platform and regulatory authorities.

#### 4.2.3. Private consultancies

Private consultancies are also key role players in the CI platform. Water Monster is a mobile application that has been developed by a private consultancy and digitally captures field data for water samples, together with sampling photos (electronic supplementary material, table S1, mobile application no. 4). Included is the MiniSASS webpage and related database, developed by Groundtruth [97,98] and its related MiniSASS mobile application [97] managed by SAIAB (The South African Institute for Aquatic Biodiversity).

#### 4.2.4. Non-governmental organizations

Stakeholder knowledge and expertise that is not generally thought of as ‘scientific’ but is widely accepted as indigenous and local, can lend credibility and transparency to scientific studies. Non-governmental organizations (NGOs) such as the Treasure Karoo Action Group (TKAG) is a dominant South African NGO that allows for the flow of information on UOG extraction activities between citizens, science and government. It aims to raise awareness about UOG, advocate for the people, promote accountability and fair decision-making practices. The TKAG has delivered critical reviews in parliament and achieved the introduction of UOG extraction moratoria [99]. FrackFree SA is another NGO that also provides links to news and information on UOG extraction in South Africa.

While the TKAG, FrackFree SA and other civil groups provide platforms for information on the latest UOG extraction-related news, there is still a gap in the critical flow of key information between the community, the UOG extraction companies, and the government, which is needed to ensure regulatory compliance and citizen science. This is where a CI platform run by a designated institution (e.g. a university, the CSIR or a private consultancy), which links to a groundwater fracking mobile application, can provide a vehicle for citizen science to play a role in ensuring regulatory compliance. A groundwater fracking mobile application does not yet exist in South Africa, and it is this specific gap that this study aims to address.

**Figure 2.**
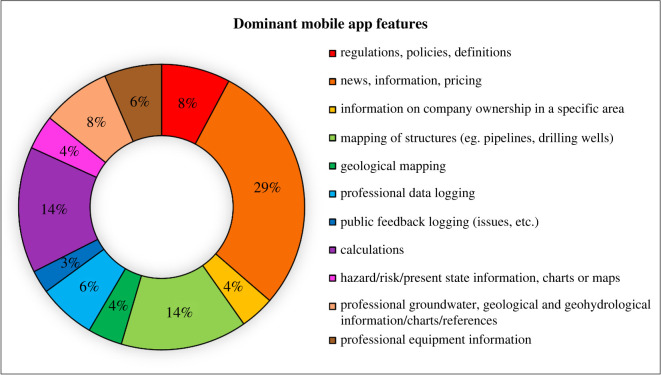
Mobile application features most dominant among the 49 mobile applications related to UOG extraction.

For the development of a proposed mobile application in the South African context, we identified and reviewed approximately 49 mobile applications commonly used by both the public and oil and gas industries internationally (see electronic supplementary material, table S2). These mobile applications have various functionalities and have been grouped into the following nine feature categories: regulations, policies, definitions; news, information, pricing; information on company ownership in a specific area; mapping of structures (e.g. pipelines, drilling wells); geological mapping; professional data logging; public feedback logging; calculations; and hazard/risk/present state information, charts or maps (See electronic supplementary material, table S3). Figure 2 shows the features of the 49 existing mobile applications after a detailed review (see electronic supplementary material, table S4).

### 4.3. Structure of the unconventional oil and gas mobile application

In a country with no prior experience of onshore UOG extraction, success in resource governance is highly dependent on including the broader society for the generation of knowledge and relative decisions [11]. A mobile application and a related platform that aims to protect groundwater resources during UOG extraction can perform this function.

To ensure the flow of critical information across one system, the mobile application can function as an information resource and a data logging mechanism [86,87,90,100,101]. The mobile application should contain an external outward-facing platform that is visible to the general public and an internal platform that is visible only to the internal platform stakeholders. The internal stakeholders of the CI platform should ideally consist of academic institutions, parastatals, private companies and NGOs. The internal stakeholders that make up the internal stakeholder platform group should be trustworthy and impartial [22] and should have various authorizations and capabilities on the map, as well as responsibilities. The internal stakeholder platform group is ultimately responsible for the overarching validation and relaying of information within the mobile application. They would be able to submit data, verify data and manage certain sections of the application to ensure that critical information is generated and communicated to all stakeholders impacted by UOG extraction, as well as to ensure regulatory compliance. This is to protect proprietary information [29,68] while still promoting transparency, credibility and the flow of critical information. The regulatory authorities, UOG companies, citizen stakeholder groups and anyone else who would like to query data, but who are not necessarily impartial, would form part of the external stakeholder platform group [20]. The external stakeholder platform group will also have various authorizations, capabilities and responsibilities.

**Figure 3.**
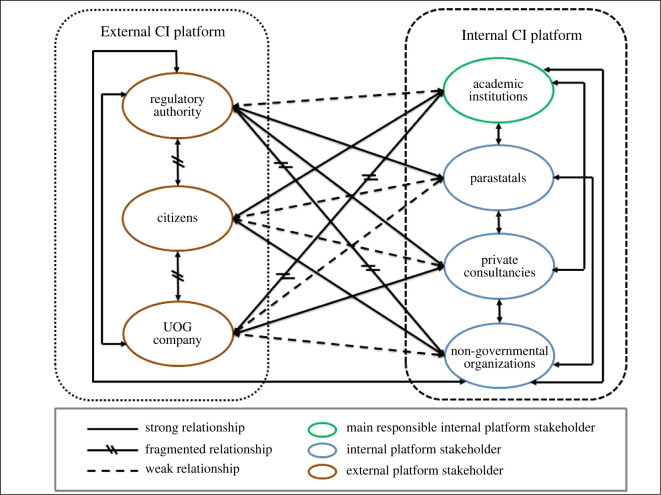
Mapping of stakeholder relationships.

The relationships between the external platform stakeholder group and the internal platform stakeholder group are mapped out in figure 3. This arrangement of stakeholders will ensure a mediated information infrastructure for the mobile application [102]. The mapping of these relationships is based on three methods of relationship analysis (electronic supplementary material, table S5). This includes traditional interest and influence relationships, social network analysis and conflict mapping, which guided the design of the mobile application stakeholder platforms.

Table 1 explains the authorizations of different functions on the mobile application for different stakeholders in terms of who can access certain functions and who will be able to log data and who is responsible for the verification of these data and relaying the information via the mobile application.

**Table 1.** Authorization, input and relaying of information via the mobile application (where ▴= regulatory authority; ▪= CI platform; ♦= UOG company; ●= citizens).

application aspects		access	logging of data	verification and relaying
legal information				
authorizations		▴ ▪ ♦ ●	▴	▪
UOG regulations		▴ ▪ ♦ ●	▴	▪
technical information				
UOG extraction				
schedules	*drilling schedules*	▴ ▪ ♦ ●	♦	▪
	*transport schedules*	▴ ▪ ♦ ●	♦	▪
	*sampling schedules*	▴ ▪ ♦ ●	▪ ♦	▪
water quality and quantity				
borehole monitoring data		▴ ▪ ♦ ●	▪ ♦ ●	▪
fracking well monitoring data		▴ ▪ ♦	▪ ♦	▪
maps				
towns		▴ ▪ ♦ ●	▪	▪
UOG extraction areas		▴ ▪ ♦ ●	▪	▪
permits and moratoria		▴ ▪ ♦ ●	▪	▪
watersheds		▴ ▪ ♦ ●	▪	▪
geographical and UOG travel routes		▴ ▪ ♦ ●	▪	▪
groundwater vulnerability maps		▴ ▪ ♦ ●	▪	▪
baseline data				
geology		▴ ▪ ♦ ●	▪ ♦	▪
seismicity		▴ ▪ ♦ ●	▪ ♦	▪
water quality		▴ ▪ ♦ ●	▪ ♦	▪
water quantity		▴ ▪ ♦ ●	▪ ♦	▪
continuous monitoring data				
seismicity		▴ ▪ ♦ ●	▪ ♦	▪
water quantity		▴ ▪ ♦ ●	▪ ♦	▪
water quality		▴ ▪ ♦ ●	▪ ♦	▪
incidents				
accident		▴ ▪ ♦	▴ ▪ ♦ ●	▪
legal		▴ ▪ ♦	▴ ▪ ♦ ●	▪
incident outcomes		▴ ▪ ♦	▴ ▪ ♦ ●	▪
reports				
incident mapping		▴ ▪ ♦ ●	▪ ♦	▪
incident regulatory compliance		▴ ▪ ♦ ●	▴ ▪ ♦ ●	▪
borehole monitoring regulatory compliance		▴ ▪ ♦ ●	▴ ▪ ♦ ●	▪

**Figure 4.**
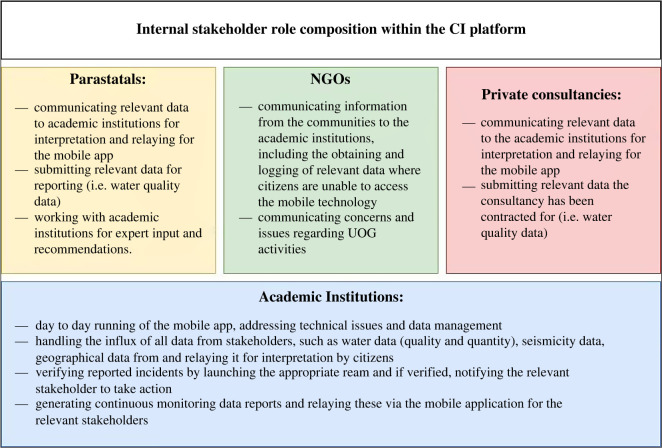
Proposed internal stakeholder structure within the civic informatics platform.

Using the information discussed above regarding the various stakeholders that could form part of the CI platform, figure 4 explains the responsibilities of the different internal stakeholders of the CI platform. The overarching internal stakeholder that would be responsible for the flow of information to citizens, would be the academic institutions. As discussed in the identification of regulatory issues, a lack of transparency and the need for an impartial or independent platform are crucial [22]. An academic institution consists of many departments that can handle various aspects of incoming data. For example, information technology departments could tackle the debugging and day-to-day smooth running of the technology of the mobile application, while a department focusing on environmental management or a geology department can verify data accuracy in their respective fields.

Parastatals could take responsibility for the communication of data to academic institutions in the case where they are contracted to do so. For example, should the CSIR be given a project to do a strategic assessment and report, the resulting report will be communicated to the relevant academic institution for relaying into the mobile application, ensuring access and communication to authorized viewers. Parastatals will also provide expert opinions and expertise where required by academic institutions [11]. These stakeholders will also provide direct links to government databases, such as the National Groundwater Archive (NGA). This specific group has strong relationships with both academic institutions and regulatory authorities [11] (figure 3), making them the preferred communication channel.

Private consultancies that are contracted for certain parts of the UOG extraction process, for example, the monitoring of borehole water levels in particular areas [97], will submit these data to the academic institution for interpretation and relaying into the mobile application.

Non-governmental organizations can be the primary link between citizens and academic institutions. Where citizens lack access to technology for gaining access to information on the mobile application, as well as reporting incidents, the NGOs should function as the approachable stakeholder to do so. Moreover, the NGOs will communicate general community concerns and issues with data should there be any.

### 4.4. Features of the unconventional oil and gas mobile application

The most important features that must be included in the design of the mobile application to protect groundwater can be seen in electronic supplementary material, table S6. These recommended features are based on a critical analysis of the existing issues in UOG extraction regulatory compliance and enforcement. Important proposed mobile application features include information on drilling and transport schedules, groundwater and seismic monitoring data, mapped geographical information, institutional baseline data and information on incidents and reports. The electronic supplementary material, table S6 also contains detailed explanations for the inclusion of these features in the proposed mobile application.

### 4.5. Information flow within the mobile application

**Figure 5.**
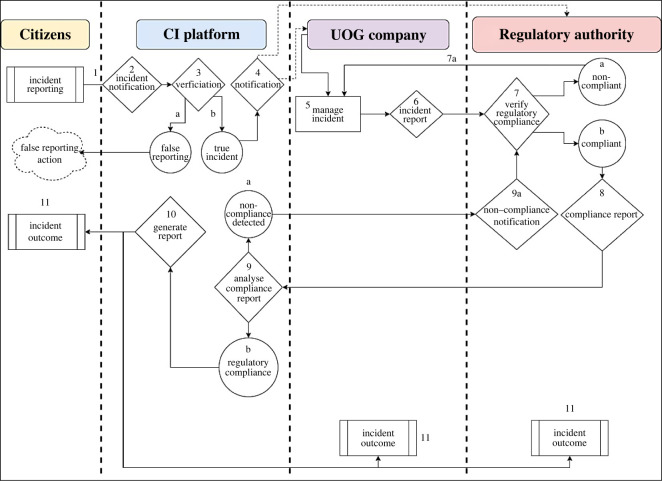
An example of the flow of information within the mobile application in the case of a citizen reporting an incident.

The flow of information through the mobile application will be determined by the nature of the specific function that is being used. The mobile application will provide the reporting stakeholder with a variety of options to choose from. Figure 5 shows a stepwise example of the logging of an incident report.

In the case of an incident that may have been logged by a citizen or UOG company, an incident notification (2) will be generated to notify the relevant internal platform stakeholder (figure 5). The internal platform stakeholder will alert the team responsible for verifying the incident. If the incident report was false (3a), then false reporting action will be taken upon the offender. If a valid incident was detected (3b), the regulatory authority will be notified (4) and the UOG company must adhere to the necessary regulations and manage the incident (5). The incident management action must be reported to the regulatory authority (6) responsible for compliance monitoring and regulatory compliance must then be verified (7). If the incident was not managed according to regulations (7a), the UOG company must adapt and manage the issue in compliance with regulations (back to 5). Should the incident be managed according to regulations (7b), a report on management compliance must be generated (8). This report will then go to the company and the relevant CI platform stakeholders who will then verify if compliance was indeed achieved (9). Should compliance not be achieved (9a), the relevant platform will send feedback to the regulatory authority to revisit the incident and take appropriate action to ensure compliance (back to 7). The cycle will repeat itself where the regulatory authority will revisit the incident and check for regulatory compliance until compliance is achieved. Once compliance has been achieved and the incident has been certified as being compliant with regulations by the CI platform (9b), the platform will generate a publicly interpretable report (10), which will be available under incident outcomes for citizens, the UOG company and the regulatory authority (11).

**Figure 6.**
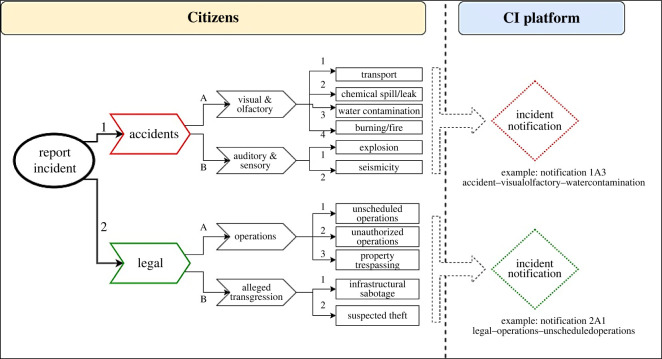
The layout and selection options an individual would be presented with should they report an incident.

The layout and options that a member of the public would see on the external outward-facing platform if they want to log an incident, can be seen in figure 6. A hypothetical example could be as follows. A citizen living next to the UOG extraction site hears an unfamiliar sound, goes out and witnesses a group of individuals running through the UOG extraction site spray-painting on the outbuildings. The citizen would then open the mobile application and select the ‘report incident’ feature. Once opened, the individual would be presented with the options ‘accident’ (1) or ‘legal’ (2), where ‘legal’ refers to law-related incidents (i.e. suspected breaking of laws) (2). Thereafter, the individual would have the option of selecting ‘operations’ (A) or ‘alleged transgression’ (B), where they would select ‘alleged transgression’ (B). Thereafter, they have the option of selecting ‘infrastructure vandalism’ (B1) or ‘suspected theft’ (B2). Once infrastructure vandalism is selected, the internal CI platform, UOG company, and regulatory authority would receive an incident notification ‘2B2’. The sequence of events depicted in figure 5 would be followed accordingly.

### 4.6. Limitations

The following are limitations that we foresee for the mobile application and should be addressed if the mobile application is to be widely used.

#### 4.6.1. Access and technology

Suitable cell phone network coverage is necessary for the smooth operation of the mobile application, to ensure GPS accuracy and data syncing. Areas such as the remote Northern Cape districts are notably rural and lack proper network coverage by most network providers (electronic supplementary material, figures S1–S5). Furthermore, the mobile application requires the use of a smartphone, implying that citizens must be able to afford these devices and the data required to access the information and maps on the mobile application. This inadvertently excludes rural communities and lower-earning citizens. With a growing divide between rich and poor, the exclusion of certain communities could result in a lack of participation.

The mobile application would therefore be useful for middle- to high-income citizens, but areas with no network coverage and lack of access will need to be addressed if the mobile application is to be a success. To address the possible limitation of access to mobile devices and data, NGOs could potentially log this information for citizens unable to log it themselves.

#### 4.6.2. Personal perspectives

As per Willems *et al.* [14], a large portion of survey respondents in a UOG development earmarked community did not understand fracking. This poses the limitation that citizens may not use the mobile application as they might not think fracking will affect them personally. There is also the possibility that community members may fear being tracked or traced through the mobile application and may not understand how their details would be protected.

#### 4.6.3. Depth of design

The mobile application considers only the most prominent issues in regulatory compliance for the protection of groundwater resources. A detailed analysis of aspects that must be included in the mobile application and the roles and responsibilities of each stakeholder group must be done to ensure that all the relevant aspects that stakeholders should be able to report on are covered by this mobile application.

## 5. Conclusion

Developing countries such as South Africa experience exponential population and industrial growth, which requires water and energy. South Africa is also energy-constrained. The country currently depends on coal for over 90% of its electricity production [15] but these reserves are dwindling [103,104]. The country is therefore considering unconventional oil and gas (UOG) extraction as an additional energy resource to improve its energy security. UOG extraction can, however, have serious environmental impacts, of which the most significant is the impact on water resources, specifically groundwater. UOG extraction requires large volumes of water, and most of South Africa's water is already allocated. Climate change variability has made groundwater a critical resource in South Africa [7,13]. Regulations must therefore be developed *and* enforced to minimize the potential impacts of UOG extraction on groundwater. Internationally, the most crucial regulatory shortcomings that must be addressed for groundwater resource protection during UOG extraction are the need for baseline data, managing the high volumes of water used and waste produced, and requiring public information disclosure of data that is presented in an understandable format and is transparent [13,25,31,33,36,52]. South African groundwater experts predominantly highlight the importance of baseline studies, regulatory enforcement, transparency, accuracy and the need for improved public information disclosure and public input [22]. A tool that can serve as an interface between the science of fracking and the protection of society and natural resources, and that can ensure policy implementation via the enforcement of regulations, is therefore required.

With impartiality and transparency in the enforcement of regulations being key concerns in South Africa, and with a fragmented governance system that fails to monitor groundwater resources and enforce its regulations properly, we propose a CI tool in the form of a mobile application that can be used to assist in the monitoring of groundwater resources and the enforcement of regulations. The mobile application could address country-specific gaps and the concerns of South African groundwater experts by providing:

— a method to guarantee the responsible use of publicly disclosed information,
— ensuring disclosed information reported by UOG companies is accurate and specific,
— allowing for input from the public, and
— disclosing information in an understandable manner and language.

This study provides a starting point for the development of a UOG extraction mobile application that is aimed at protecting South African groundwater resources. It explains the structure of such a mobile application, the stakeholders who would have access and specific authorizations within the mobile application and the features that a potential mobile application of this nature could include to assist in the monitoring and protection of groundwater resources during UOG extraction.

A mobile application that ensures the consistent flow of key information between relevant stakeholders, that has carefully controlled access, that has an overarching responsible platform stakeholder to verify data and provides access to the public, could assist in bridging the regulatory gaps in groundwater protection during UOG extraction. While many mobile applications relating to UOG extraction are used in countries that already extract UOG resources, none of them use CI to address both groundwater monitoring and UOG extraction operations in a single platform. Our mobile application proposes to combine these functions to enable regulators to protect groundwater resources more efficiently during UOG extraction, while simultaneously enhancing transparency in the UOG industry through an all-inclusive technology.

## Data Availability

The underlying data that was used in this study is available in the electronic supplementary material [105].
